# Comparative Medicine
**Cours de Médecine Comparée* (Introduction). Par P. Rayer, Membre de l'Institut (Académie des Sciences) et de l'Académie Impériale de Médecine, Professeur et Doyen de la Faculté de Médecine de Paris, Médecin Ordinaire de l'Empereur, etc. Paris: Baillière. pp. 52. 1863.


**Published:** 1863-07

**Authors:** 


### Art. V.—COMPARATIVE MEDICINE.*

We copy from the cover of an introductory lecture by M. Rayer, not only its title but the most important titles of its

* *Cours de Médecine Comparée* (Introduction). Par P. Rayer, Membre de l'Institut (Académie des Sciences) et de l'Académie Impériale de Médecine, Professeur et Doyen de la Faculté de Médecine de Paris, Médecin Ordinaire de l'Empereur, etc. Paris: Baillière. pp. 52. 1863.

author; in order that, if we have any readers who are not fully acquainted with his fame, they may see how distinguished is the position of the man who brings his great reputation to the aid of endeavours to establish medicine upon a basis co-extensive with physiology, and to find in the disorders incident to the inferior creation, facts or principles that may elucidate the disorders incident to mankind. In this introductory lecture, M. Rayer has, it is true, very much to say for himself, and very many reasons to assign for the existence of the chair he is about to fill. We think, however, that he states his case not altogether without some inward consciousness of its inherent weakness, and in a manner, *quasi* apologetic, that exhibits some distrust of his own elaborate reasoning. We will first sketch an outline of his pamphlet, and then briefly state the reasons that lead us to doubt the present practical utility of his teaching.

Being a Frenchman, M. Rayer commences, of course, with a definition. He would have comparative medicine include within its domain all organized beings, and draw its materials from two chief sources, comparative pathology and experimental pathology, that is to say, from natural and artificial maladies. His object is, therefore, to form a school in which human medicine shall be considered in its relations to the medicine of the animal kingdom, in order that the former may be enlarged and elucidated by the latter.

So much being premised, the lecturer next devotes a large portion of his discourse to the past history of the first element in his new science, the comparative pathology, or the natural maladies of the animal creation. He cites Littré's translation of Hippocrates, to show that the father of medicine did not disdain to study the diseases of domestic animals, and he passes on to Celsus and Galen, dwelling with fond satisfaction upon the endeavours of the latter to illustrate human diseases by the dissection of dead animals, among which "*un certain singe*" is brought into conspicuous prominence. From Galen he passes at a single leap to the latter portion of the 17th century, to the labours of Ramazzini, Larcisi, and Vicq d'Azyr, who appear to have done no more than observe the simultaneous or consecutive occurrence of epizootic and epidemic disorders. After them, Erhard Brunner, a pupil of Stahl, discussed in 1695, the relative frequency of human and bestial diseases, and Bergmarn, a pupil of Blumenbach, published in 1804, his *Primæ lineæ pathologiæ comparatæ*. Passing over one or two works of small importance, we arrive at Heusinger, and his *Nosographie comparée*, published in 1844. With this writer, the independent literary history of the subject terminates, and M. Rayer proceeds to enumerate the societies, medical and philosophical, whose efforts have been directed, from time to time, to the encouragement of research into the diseases of the brute creation, and their relations to those of man. From such efforts sprang a variety of prize essays, by authors either altogether obscure or remembered only by other writings; but according to M. Rayer, these prize essays were the means of giving an impulse to the study of their respective subjects, and were the original causes of such works as those of MM. Andral and Gavarret on hæmatology. Various writings on calculus, parasites, and helminthology, human and comparative, are also mentioned in terms of laudation, but with the placid ignorance of a French *savant* concerning all things English, Mr. Youatt's researches upon rabies are passed over without the slightest notice.

The next section of the discourse is devoted to the history of experimental pathology, and contains a brief account of the most important results obtained by the artificial production of diseased conditions in animals. Marvellous to relate, M. Rayer commences his enumeration of the past labourers in this department with the name of our countryman Lower, and passes on, with a glance at Baglivi, van Swieten, and others, to the works of Magendie, Flourers, Claude Bernard, Longet, Cruveilhier Jobert (de Lamballe), Chossat, and Orfila. Virchow is barely mentioned in the text; and one or two other Germans in the notes; but for which exceptions, M. Rayer's readers might suppose experimental pathology to be a science unknown beyond the limits of the French empire, and incapable of expression in any but the French language.

Having thus indicated the sources from which his facts will be derived, M. Rayer proceeds to say that the field before him is too vast to be at first exhaustively examined. He purposes to select the most important subjects, and to enlarge his course by degrees. For the present year he will occupy himself with the consideration of the maladies transmissible from the lower animals to man; hoping, by tracing them to the beast from which they spring, to obtain increased knowledge of their fundamental characters and nature, of the modifications impressed upon them by different species, and of the principles that should regulate their treatment. Of such diseases, he enumerates cow-pox, rabies, farcy, and the varieties of mange, or itch, as the most important; the inflammations and affections of the genital organs of animals are also mentioned; and the shelter afforded by certain animals to the earlier stages of tænia.

There is a homely French proverb, of which the pages of M. Rayer's lecture have continually reminded us—"*Le jeu ne vaut pas la chandelle.*"

We should be the first to welcome an enlarged application of hygienic principles to the preservation of animal life. In this country, where cattle breeding is conducted with unparalleled success and care, we are nevertheless dependent for a great portion of our food supply upon imported flocks and herds, too often the bearers of destructive and contagious diseases to our own domestic animals. The sources of origin of cattle murrains, and the methods by which they might be prevented from arising or from spreading, are matters worthy of the most earnest and thoughtful consideration. As a rule, veterinary practitioners cannot be expected to possess the knowledge, or the grasp of mind, required for the study and application of great general principles, and hence the hygiene of the brute creation may properly and worthily occupy the attention of medical philosophers. But to seek from brutes an elucidation of the diseases of mankind appears to us the dream of a crazy enthusiast, better fitted for a professorship in the University of Laputa, than to be a member of the Institute of France.

We must, in the first place, demur to M. Rayer's attempt to quote Hippocrates and Galen as the founders of his project. Galen would not have rested his human pathology upon the dissection of apes, if the dissection of men had been within his power.

Without resting, however, upon an objection so unimportant as this, we will proceed to state our reasons for attaching but slight value to the projected course of comparative medicine. We think that the pathology of the brute creation must necessarily remain too much in the rear of human pathology to elucidate the latter in any material degree; and that veterinary therapeutics could only serve to mislead the practitioner of human medicine.

In the first place, the great stimulus to scientific research in all departments of inquiry is the value of the object sought to be attained; and just in proportion to this value will be the number and capacity of the labourers. The preeminent value of human health and life will always attract a large number of men of the highest order of mind to the study of human pathology, and the possibility of obtaining the intelligent co-operation of the patient in the elimination, or right estimation, of disturbing influences, will always greatly facilitate observation of the natural history, or essential characteristics of human diseases. In the brute creation, however, where the value of health and life is reduced to the limited money value of the animal, or even to its diminished money value after severe illness, or lingering convalescence, *minus* the absolute value of its carcass, there are no inducements to attract skilled labour into so barren and unremunerative a field. M. Rayer's own devotion to science may lead him to dabble in veterinary medicine; but, as long as there are but twelve hours in a day, and as long as human beings are of more value than beasts, the majority of skilled and scientific practitioners will confine themselves to human patients, and the majority of veterinary practitioners will be men of inferior education and limited attainments. It follows, we think, that the great bulk of occurring instances of bestial disease will fall under the observation of men little accustomed to ascend from particular instances to careful generalization, and that medical philosophers will only bestow an occasional and spasmodic attention to special epizootic visitations; an attention in which, from want of familiarity with the ordinary aspects of their new patients, they will be more likely to fall into error than to stumble over truth. As a matter of fact, we believe the advances of recent years in veterinary medicine have been chiefly obtained by the application, to the lower animals, of the principles of human pathology and therapeutics; and, in the nature of things, the aid thus afforded by the greater to the less, hardly admits of being returned in kind. It must be remembered that the diagnosis of bestial is far more difficult, and consequently, in a general way far less exact, than that of human ailments, even independently of the consideration that it is usually in less able hands, while the essential and accidental phenomena of any particular case must always be extremely difficult to separate, in consequence of the inability of the sufferer either to respond to questions, or to understand and promote the objects of treatment.

For the second element, the experimental pathology, we have not much more respect than for the first. Physiologically considered, the lower animals, in the graphic words of Cuvier, may be regarded as experiments performed by nature for our instruction. They often manifest functions under their simplest conditions, and dependent only upon their essential organs. They lead us, by natural gradations, from the simpler to the more complex; from the lowest forms of conscious life to the intricate organism of man.

Experimental pathology, however, is a pursuit of far more doubtful value. In the first place, the conditions of human disease cannot be precisely imitated by artificial injuries. In the next place, the nervous system and moral feelings of man exert a powerful influence upon his disorders and his efforts at repair. With a few exceptions, fewer perhaps than are generally made, we believe that all the fruits of experimental pathology might have been obtained without its aid, by patient and careful observation of the sick, and that experimenters seldom gain more than a little time in the discovery of some particular fact. Whether the time be worth gaining, at the expense of cruelty and at the risk of error, must be decided by the nature of the information sought, and the urgency with which it is required. We think there can be no question that comparatively few experiments, when regarded from this point of view, could be justified by right minded men; and the experimental pathologists lauded by M. Rayer, have very seldom gained advantages at all equivalent to the misery they have caused. Take the single example of M. Chossat (de Genève). If we remember rightly, this pseudo scientific miscreant starved hundreds of animals to death, and watched and chronicled their lingering agonies, without enlarging science by a single new principle, or even by a single important fact. Instead of a niche in the temple of fame, we should feel disposed to award him such discipline as a costermonger who had been caught skinning live cats would receive from a London police magistrate. By his side, in M. Rayer's enumeration of worthies, are placed the names of some of the professors at that veterinary college at Alfort, the manifold cruelties of which have lately called forth the condemnation of the civilized world. Comparative medicine will need to bring forth fruits, to rescue itself from the dishonour of such alliances as these.

We pass on to M. Rayer's hope to obtain by a more extended study of disease a more accurate knowledge of its essential nature, and important guidance in treatment. The last clause of the sentence seems to us to contain a concise embodiment of the error that lies at the root of most quackery; an error that a few practitioners in this country share with many in France, and with the great bulk of the unprofessional public all over the world. The aim of medicine is to treat patients, not to treat diseases; and the personal peculiarities of the sufferer are far more important, as therapeutical indications, than any or all of the names in the *Statistical Nosology*. A knowledge of what M. Rayer calls the essential nature of disease may sometimes be useful in its prevention, but seldom or never in its cure; and there is no more conspicuous evidence of the absence of "the gift of healing," than a prescription addressed to the pneumonia, or the peritonitis, instead of to A. or B. Quacks and nostrum vendors of all sorts; druggists who make "bilious pills;" and old women of both sexes who swallow them, believe, or profess to believe, that medicines are antidotes to some specific evils; that diseases, in the happy phrase of Miss Nightingale, are "distinct entities, like dogs and cats," requiring to be banished by appropriate agents, and that the complaint, instead of its victim, is the proper object of the skill of the physician. Errors so deeply rooted in the public mind are imbibed in almost every English nursery, and are often only partially banished by an English medical education. But in France they reign supreme. They give their colour to the great bulk of French medical literature; they animate and sustain French vivisection; they inspired the physician who exclaimed triumphantly, over his hapless patient, *Il est mort guéri;* and they find their culminating point in the serious proposal, by the physician to the Emperor, to seek, from the complaints of calves and jackasses, some fresh knowledge concerning those of his countrymen.

---

